# *In Vitro* Comparison of the Effectiveness of Chlorhexidine and Two Calcium Hydroxide Formulations on *Enterococcus Faecalis*

**Published:** 2008-07-10

**Authors:** Mohammad Reza Sharifian, Noushin Shokouhinejad, Marzieh Aligholi, Mohammad Emaneini, Arash Katebi, Hadi Assadian

**Affiliations:** 1 *Department of Endodontics, Dental School, Dental Research Center, Tehran University of Medical Sciences, Tehran, Iran*; 2 *Department of Endodontics, Dental School, Dental Research Center, Tehran University of Medical Sciences/ Iranian Center for Endodontic Research, Tehran, Iran*; 3 *Instructor of Microbiology, School of Medicine, Tehran University of Medical Sciences, Tehran, Iran*; 4 *Department of Microbiology, School of Medicine, Tehran University of Medical Sciences, Tehran, Iran*; 5 *General practitioner, Tehran, Iran*; 6 *Department of Endodontics, Dental School, Tehran University of Medical Sciences, Tehran, Iran*

**Keywords:** Calcium Hydroxide, Chlorhexidine, Enterococcus Faecalis, Root Canal Medicaments

## Abstract

**INTRODUCTION:** The aim of this *in vitro *study was to compare the effectiveness of three intracanal medicaments in disinfecting the root canal and dentin of experimentally infected human teeth with *Enterococcus faecalis *(EF).

**MATERIALS AND METHODS:** One hundred extracted human single-rooted teeth were used. After root canal preparation, teeth were mounted in epoxy resin. Following sterilization, the teeth were infected for 28 days with EF. Then root canals were filled with one of three different disinfectants: viscous 2% Chlorhexidine (CHX), calcium hydroxide paste (CH) or a mixture of CH and CHX (n=30 in each group). Antimicrobial assessments were performed at 1, 3 and 7 days (n=10 in each time period). Microbial samples were obtained from root canals before and after the experiment. Also dentin samples were examined. The data was analyzed using Two- Way ANOVA test.

**RESULTS:** The findings showed that there was no difference between experimental groups at different time periods. The mixture of CH/CHX in 7 days was able to eliminate EF completely from root canal system. The most elimination of EF was from dentinal tubules.

**CONCLUSION:** According to the results of this *in vitro *study, viscous 2% CHX, mixture of CH with distilled water and 2% CHX are all effective disinfectants.

## INTRODUCTION

The main purpose of root canal therapy is to eliminate microorganisms and their products from the root canal system as well as to prevent re- infection. Although mechanical canal preparation plays an important role in root canal cleaning, it is not capable of thoroughly eliminating microorganisms from the complex root canal system. Investigators have noted that bacteria in instrumented, unfilled canals can multiply and reach their pretreatment numbers in 2 to 4 days (1). In such cases dressing of root canals using antimicrobial medicaments are advocated (2,3). *Enterococcus faecalis *(EF) is a resistant microorganism that etiologically plays an important role in persistent periapical lesions. Studies have shown that EF is the most prevalent microorganism populating in root canals with therapy-resistant lesions (4). Several papers demonstrated its resistance to calcium hydroxide (CH); the most common intracanal medicament used in endodontic treatments (5,6).

Chlorhexidine gluconate (CHX) has a wide- spectrum antimicrobial activity against gram positive and gram negative microorganisms, bacterial spores, lipophilic viruses, and yeasts (7). This agent has the acceptable biocompatibility and feasibility to be used as an inter-appointment dressing (8). CHX acts as an antimicrobial reservoir; so that it is absorbed by hydroxyapatite then it is released from the tissues when its concentration falls (9). Hence, in order to achieve a broad-spectrum and long lasting antimicrobial effect, it is suggested that CH powder be mixed with antimicrobial irrigants such as CHX instead of water (10). The mixture of CH and CHX compared with CH and water in order to enhance its effectiveness is controversial.

"Consepsis V" is a viscous form of 2% CHX manufactured for endodontic intracanal medication. To date, there is only one published study (11) concerning the antimicrobial effect of "Consepsis V" and CH paste which has not evaluated the ability of dentin disinfection of these medicaments. Therefore this *in vitro *study was designed to evaluate the antimicrobial efficacy of "Consepsis V" and CH mixed with different vehicles (distilled water or 2% CHX) on human teeth infected with EF.

## MATERIALS AND METHODS

A total of 100 extracted human single-canalled roots without caries, microcracks or root-end curvatures were included in this study. As a pilot dentin shavings were prepared from 5 samples to reassure the penetration of EF into the dentinal tubules. After culturing, presence of EF was documented in dentin shavings.

After disinfection with 5.25% NaOCl solution, the teeth were decoronated to achieve root length of 13 to 15mm. The canals were prepared using step-back technique. Preparation of the apical area was done up to size #40, then flaring the canal was performed up to size #80. Irrigation was performed between each instrument using 2 mL of normal saline, and then all the roots were mounted in epoxy resin. In order to prevent entrance of epoxy resin into the canal through apical foramen, all apices were covered with a layer of glass ionomer cement (GC Corp., Tokyo, Japan) before mounting in resin blocks. Finally 17% EDTA (Ariadent; Asia Chemi Teb, Tehran, Iran) was used for 1 min and then 5 mL of 5.25% NaOCl (Vista Dental Products, Racine, WI, USA) for 3 min to remove smear layer. Afterwards, the samples were autoclaved.

EF (ATCC 29212) was cultured in Brain Heart Infusion (BHI) agar medium. During the 28 days of study, microbial suspension was replenished in the canals every 2 days except for negative control group (n=5).

After inoculation time (28 days) sampling was performed to count EF growth. Initially, three sterile paper points #35 (Gapadent CO., LTD., Korea) were placed in each canal with 30-second intervals. Then the paper points were placed in tubes containing 1 mL of normal saline. The tubes were vortexed for 20 seconds to distribute microorganisms within normal saline. From the serial samples, 10**-1**, 10**-2**, and 10**-3 **dilutions were prepared. After that, 0.1 mL was taken from each dilution and placed on culture medium and smeared by an L-shaped sterile bar. EF counting was done in BHI agar culture medium.

After CFU1 counting, intracanal medicaments were used in experimental groups containing 90 teeth (10 teeth in each group) in the following order:

- 2% CHX (Consepsis V, Ultradent Products Inc. South Jordan, UT, USA) for 1 day

- 2% CHX for 3 days

- 2% CHX for 7 days

- Mixture of Ca(OH)2 (Merk, Darmstadt, Germany) /distilled water for 1 day

- Mixture of CH/distilled water for 3 day

- Mixture of CH/distilled water for 7 day

- Mixture of CH/2% CHX for 1 day

- Mixture of CH/2% CHX for 3 day

- Mixture of CH/2% CHX for 7 day

Normal saline was placed in the canals of positive control group (n=5).

In negative control group (n=5), microbial suspension was not inoculated in the canals after sterilization.

After placing intracanal medicaments and sealing the orifices with temporary filling material, the samples were incubated at 37ºC for the whole experiment period. After 1, 3 and 7 days in above mentioned groups, intracanal medicaments were rinsed with 5 mL of normal saline and another sample was taken from the canals referred to as CFU2. Irrigation with 5 mL of normal saline was also performed in the positive control group.

**Table 1:** Antibacterial activity of intracanal medicaments (IM) against intratubular bacteria in different time periods (n=10 specimens in each subgroup)

IM ^a^	Time (Day)	Growth	No Growth
	1	7	3
CHX ^b^	3	5	5
	7	5	5
	1	5	5
CH^c^/DW^d^	3	5	5
	7	7	3
	1	6	4
CH/CHX	3	3	7
	7	1	9
PC^e^		5	0
NC^f^		0	5

**a:**Intracanal medicament, **b:**2% Chlorhexidine, **c:**Ca(OH)2, **d:**Distilled water, **e:**PPositive Control, **f:**Negative Control

After preparing CFU2 from the canals, the teeth were taken out of resin blocks. The dentin chips were prepared by shaving the dentinal walls from the outside of the root surface inwards with a sterile round bur. Thus a thin layer of dentin remained between the external wall of the root and intracanal space. Then dentin chips were placed in tubes containing BHI agar. After incubation for 48 hrs in 35°C, tubes were evaluated for growth of microorganisms by turbidity testing and positive cases were registered.

Thereafter to guarantee that environmental contamination has not occurred and to see whether there are any remaining microbes in the tubules aliquots were taken from turbid media and aerobically cultured on BHI agar. After incubation time, gram staining and diagnostic tests were performed to detect microbial entities. Data was analyzed using Two-Way ANOVA test.

## RESULTS

**Figure 1:**
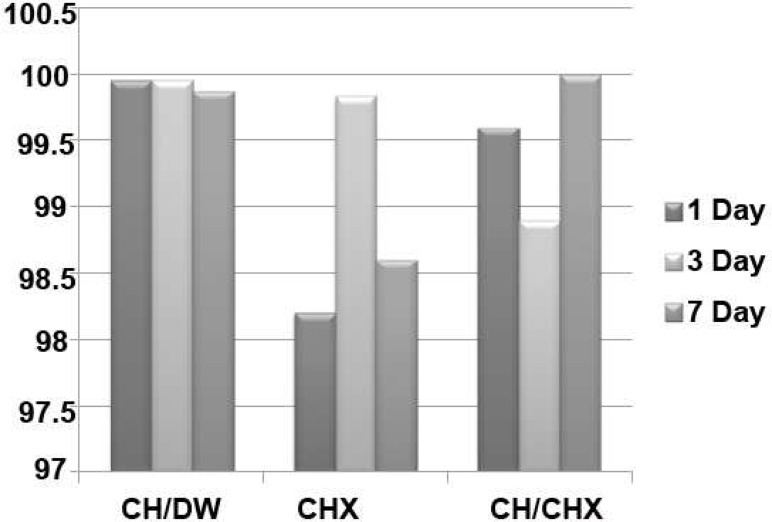
Mean decrease of *Enterococcus faecalis *colony counts from the canal space

In negative control group, the samples taken were all negative. On the other hand, in positive control group 38% decrease in EF count was observed. The results for the mean decrease of EF counts from canal space are presented in Figure 1.

Statistical analysis showed that neither the type of intracanal medicaments nor the time intervals were significantly effective in decreasing EF colony counts from the canal space (P>0.05). However, in the mixture of CH/CHX group, EF was completely removed after one week from the canal space in all the samples.

The dentin shavings taken from the samples were also evaluated for the presence of EF. The results showed that all three medicaments were able to significantly eliminate intratubular microorganisms (Table 1). Although the most prominent value of decrease in number of intratubular microorganisms was seen in the mixture of CH/CHX group after 1 week, there was no significant difference among the three medicaments in dentin disinfection in different time intervals (P>0.05).

Irrigation by distilled water *per se *could not disinfect dentinal tubules in any of the positive control samples. Dentin shavings taken from negative control samples were free from EF.

## DISCUSSION

*Enterococcus faecalis *was selected due to its high prevalence in therapy resistant cases and root treatment failures (12-15). As mentioned this microorganism is resistant to antibacterial effects of CH (4,16). Four-week inoculation period was carried out for bacterial inoculation into the canals to reassure the entrance of bacteria into dentinal tubules (5).

Initially a pilot was performed to make sure EF did penetrate into dentinal tubules. Furthermore, presence of bacteria within dentin samples taken from root canal wall shavings in positive control group indicates that removal of smear layer and entrance of bacteria into dentinal tubules were done successfully. The negative results of the samples from negative control group in various stages of the study indicate the accuracy of sterilization initially and absence of sample contamination during the process.

The reason for preparation of dentin chips from outside root surface inwards was to prevent dentin samples becoming contaminated by microorganisms attached to root canal walls (innermost dentin layer of root wall).

Depletion of EF counts as much as 38% in root canals of the positive control group can be attributable to washing out of microorganisms during saline irrigation before second sampling. It can be noted that physiological saline solution alone, was not able to disinfect dentinal tubules in any of the positive control samples.

Some investigators have questioned the effectiveness of CH to disinfect the canals, reporting that even after one-week of dressing with CH, microorganisms could still be detected in canals and root dentin (17,18).

The current study indicates that CH failed to thoroughly eliminate EF in study samples. This was in accordance with Evans *et al. *(4) and other previous studies (19-24). Decrease in EF counts by over 99% after the use of CH paste indicated its effectiveness in chemical cleaning of the canals. This finding concurs with that of Shuping *et al. *(25), Gomes *et al. *(26), and Sjögren *et al. *(3).

Although CH was effectively able to eliminate microorganisms from root canal space, it should be noted that an intimate contact between microorganisms and CH only occurs in the main root canal space (particularly in straight canals with round cross-sections). This form of contact might not arise throughout complex root canal system. Also, microorganisms may penetrate dentine tubules and evade contact with irrigants (26).

Resistance of EF against CH has drawn attention to the use of other intracanal medicaments like CHX or the mixture of CHX and CH. CHX can bind to dentine hydroxyapatite due to its cationic properties (27). The bound CHX has excellent substantivity enabling it to prevent bacterial colonization within the root canal system (28). Viscous 2% CHX (ConsepsisV) was used in this study, being designed as an intracanal dressing between treatment sessions. 2% CHX *per se *was not capable of eliminating bacteria from the samples, but a mean 98.17% decrease in 1 day, a 99.87% in 3 days, and a 98.64% in 7 days shows effectiveness of this medicament. Contrary to these results Schafer *et al. *(19) showed that CHX can have a significant effect on EF after 3 days as it will penetrate dentin thoroughly. A significant difference was illustrated with CH paste and mixture of CH/CHX in removing EF.

Other investigations have also emphasized the significant effect of CHX against EF (6,19,23,26,29,30). One *in vivo *study showed that CHX gel can be used as an effective root canal disinfectant during instrumentation (31).

Several authors believe that the mixture of CH and CHX can have an inhibitory effect on CHX.

Hence, the antimicrobial efficacy of this mixture is equal to that of CH mixed with distilled water (19,32-34). CHX contains positive charge while CH is negatively charged. The decreased effectiveness of CHX may be due to the loss of biguanide proton in pH values above 10. This phenomenon can cause a significant decrease in antimicrobial efficacy and a modified reaction with bacterial surfaces due to the change in electrical charge of CHX molecules (32,34).

Conversely, other investigations concluded that CHX can render CH more potent against resistant microorganisms (23,26,35-37). Our study demonstrated that although the mixture of CH/CHX produces bacteria free canals in seven days, the results were not statistically different with the CH/distilled water mixture, concurring with several other studies (19,23,29,31-34). Also these results support the findings of Manzure *et al. *(11) which demonstrated a mean decrease in EF colonies from the root canals associated with apical periodontitis, concluding that the antibacterial efficacy of CH, CHX, and CH/ CHX was comparable.

In the present study, there was no significant difference between various intracanal medicaments and different time intervals, when dentin shavings were evaluated. These findings are in agreement with another investigation (22). Our study showed that the mixture of CH/CHX for 7 days had greater efficacy in removing EF contamination from the infected dentin, contradicting studies stating that CHX *per se *can significantly eliminate EF from contaminated dentin (19,23).

Some authors believe that CH, in order to achieve its optimal antimicrobial efficacy, has to remain within the canals at least for 1 week (2), whereas the current study showed no significant difference between 1, 2, and 7-day intervals in terms of mean decrease in EF colonies from the canals and infected dentin.

The controversy between the results of various studies can be attributed to the different methods of research. Furthermore, it is not possible to equalize *in vitro *results with clinical conditions. Increased antimicrobial agent availability, better accessibility of agents to all target microorganisms, and lack of substances such as serum albumin and exudates which deplete antimicrobial efficacy intracanal medicaments are some of the differences between laboratory and clinical settings (26,38).

According to the present study increased pH of CH is maintained even when mixed with 2% CHX (39) and therefore the mixture of CH/CHX might be effective when an alkaline environment is required to stop inflammatory resorption and carry out disinfection of the canal and dentin. However, further studies are necessary in this regard.

## CONCLUSION

Under the condition of this *in vitro *study, viscous 2% CHX and mixture of CH with distilled water or 2% CHX were all effective for disinfection of root canal and dentin contaminated with EF.
